# The Endocannabinoid System across Postnatal Development in Transmembrane Domain *Neuregulin 1* Mutant Mice

**DOI:** 10.3389/fpsyt.2018.00011

**Published:** 2018-02-07

**Authors:** Rose Chesworth, Leonora E. Long, Cynthia Shannon Weickert, Tim Karl

**Affiliations:** ^1^School of Medicine, Western Sydney University, Campbelltown, NSW, Australia; ^2^Schizophrenia Research Institute, Sydney, NSW, Australia; ^3^Neuroscience Research Australia, Randwick, NSW, Australia; ^4^School of Psychiatry, University of New South Wales, Sydney, NSW, Australia

**Keywords:** schizophrenia, endocannabinoid system, neuregulin 1, development, cannabinoid receptor 1, diacylglycerol lipase alpha, monoglyceride lipase, α/β-hydrolase domain-containing 6

## Abstract

The use of cannabis is a well-established component risk factor for schizophrenia, particularly in adolescent individuals with genetic predisposition for the disorder. Alterations to the endocannabinoid system have been found in the prefrontal cortex of patients with schizophrenia. Thus, we assessed whether molecular alterations exist in the endocannabinoid signalling pathway during brain development in a mouse model for the schizophrenia risk gene *neuregulin 1* (*Nrg1*). We analysed transcripts encoding key molecules of the endocannabinoid system in heterozygous transmembrane domain *Nrg1* mutant mice (*Nrg1* TM HET), which is known to have increased sensitivity to cannabis exposure. Tissue from the prelimbic cortex and hippocampus of male and female *Nrg1* TM HET mice and wild type-like littermates was collected at postnatal days (PNDs) 7, 10, 14, 21, 28, 35, 49, and 161. Quantitative polymerase chain reaction was conducted to assess mRNA levels of cannabinoid receptor 1 (CB_1_R) and enzymes for the synthesis and breakdown of the endocannabinoid 2-arachidonoylglycerol [i.e., diacylglycerol lipase alpha (DAGLα), monoglyceride lipase (MGLL), and α/β-hydrolase domain-containing 6 (ABHD6)]. No sex differences were found for any transcripts in either brain region; thus, male and female data were pooled. Hippocampal and cortical mRNA expression of DAGLα, MGLL, and ABHD6 increased until PND 21–35 and then decreased and stabilised for the rest of postnatal development. Hippocampal CB_1_R mRNA expression increased until PND 21 and decreased after this age. Expression levels of these endocannabinoid markers did not differ in *Nrg1* TM HET compared to control mice at any time point. Here, we demonstrate dynamic changes in the developmental trajectory of several key endocannabinoid system transcripts in the mouse brain, which may correspond with periods of endocannabinoid system maturation. *Nrg1* TM HET mutation did not alter the developmental trajectory of the endocannabinoid markers assessed, suggesting that other mechanisms may be responsible for the exaggerated cannabinoid susceptibility in these mice.

## Introduction

Schizophrenia is a chronic, debilitating mental disorder that can develop through interactions between genetic susceptibility and environmental insults (1–3). The use of cannabis can increase the risk of developing schizophrenia (4, 5), and usage rates are elevated in individuals who carry genetic risk alleles for the disorder (6). Furthermore, a single-nucleotide polymorphism associated with cannabis dependence is located in the *neuregulin 1* (*NRG1*) gene, which is a susceptibility gene for schizophrenia (7, 8). *NRG1* and its cognate receptor ErbB4 regulate the development of activity-driven glutamatergic synapse development (9), indicating that *NRG1* signalling may contribute to schizophrenia pathophysiology in the context of the glutamate hypothesis of schizophrenia. While the mechanisms by which cannabis exposure and genetic factors interact to bring about psychotic illness are unclear, it is possible that genetically induced dysregulation of endogenous cannabinoid signalling may be a contributing factor (10).

The endogenous cannabinoid (endocannabinoid) system is constituted by endogenous receptors [e.g., cannabinoid receptor 1 (CB_1_R) and cannabinoid receptor 2] and ligands that regulate neurotransmission and govern neurodevelopment [e.g., 2-arachidonoylglycerol (2-AG) and *N*-arachidonoylethanolamine] (11). Importantly, the endocannabinoid system appears to undergo changes in patients with schizophrenia. Elevated levels of anandamide, one of the main brain endocannabinoids, are present in the cerebrospinal fluid of patients with schizophrenia (12) and are inversely associated with psychotic symptoms in treatment naive patients (13). Also, mRNA expression of the predominant brain cannabinoid receptor, which mediates the psychoactive effects of cannabis, CB_1_R, is reduced in the dorsolateral prefrontal cortex (DLPFC) of patients with schizophrenia (14, 15). In contrast, we have also found elevated CB_1_R binding in the DLPFC of people with paranoid schizophrenia (16). Thus, is possible that elevated risk for developing schizophrenia following cannabis exposure may result from altered endocannabinoid signalling. Although the nature of the change to the endocannabinoid system found in adults is unclear, it is possible that the development of the endocannabinoid system could be altered in schizophrenia either as an underling disruption or occurring in response to the disease. In line with this is the fact that the endocannabinoid system undergoes significant changes around the typical time of onset of schizophrenia (e.g., adolescence to early adulthood) (17).

We sought to address two aims: (1) to chart the developmental trajectory of mRNAs involved with the endocannabinoid system in murine brain and (2) to determine whether developmental changes in the endocannabinoid signalling system may occur as a direct response to genetic susceptibility to schizophrenia. We did this by examining two schizophrenia-relevant brain regions across postnatal development (i.e., the prelimbic cortex and the hippocampus) in an established mouse model for schizophrenia, the heterozygous transmembrane domain *Nrg1* mutant mice (*Nrg1* TM HET). *Nrg1* TM HET mice display construct validity for schizophrenia, as schizophrenia patients have been found to exhibit a missense mutation in the transmembrane domain of *NRG1* (18). *Nrg1* TM HET mice also exhibit face and predictive validity for schizophrenia, as they display behavioural features relevant to the symptoms of schizophrenia (19–22), some of which can be ameliorated by antipsychotic treatment (7). Importantly, *Nrg1* TM HET mice also exhibit altered susceptibility to the behavioural and neural effects of cannabinoids (23–28), which is relevant to the increased cannabis sensitivity observed in patients with schizophrenia (29).

Previous work in our laboratory demonstrated region-specific changes in receptor binding for CB_1_R during adolescence and adulthood in *Nrg1* TM HET mice (10, 28). However, these studies did not test the extent to which these changes may have preceded adolescence [analysis of brains from postnatal day (PND) 53 onwards only] and only examined CB_1_R. This study examined the developmental trajectory of four endocannabinoid system transcripts from PND 7–161. We assessed mRNA expression of CB_1_R, as well as of the enzymes diacylglycerol lipase alpha (DAGLα), monoglyceride lipase (MGLL), and α/β-hydrolase domain-containing 6 (ABHD6). We chose these enzymes as they catalyse the synthesis and hydrolysis of the most abundant brain endocannabinoid, 2-AG. We quantified mRNA in the hippocampus and prelimbic cortex because these regions show high levels of CB_1_R expression (30) and are critically implicated in schizophrenia (31, 32). We hypothesised that there would be region-specific dynamic changes in CB_1_R, DAGLα, MGLL, and ABHD6 mRNA across development. We also hypothesised that the mRNA expression of endocannabinoids would be different in *Nrg1* TM HET mice compared to control mice—notably that the expression of these markers may be elevated in *Nrg1* TM HET mice or may occur earlier in mutant mice compared to control littermates—and that these differences may account for the altered response of *Nrg1* TM HET mice to cannabinoids (33).

## Materials and Methods

### Animals

Male and female heterozygous *Nrg1* TM^+/–^ mice (*Nrg1* TM HET) and *Nrg1* TM^+/+^ [wild type (WT)] littermates were bred as described previously (19) and backcrossed for >10 generations onto a C57BL6/JArc background. Mice were bred and housed at Australian BioResources (Moss Vale, Australia). Mice were housed with their dams in litters prior to weaning at PND 21, where they were group housed (2–4 animals per cage) in individually ventilated cages (Type Mouse Version 1; Airlaw, Smithfield, Australia; air change: 90–120 times per hour averaged; air speed: 0.12 m/s; passive exhaust ventilation system). Cages contained a wire hopper, giving the animals some limited vertical climbing opportunities, and also contained a mouse igloo (Bioserv, Frenchtown, USA) and tissues for nesting material. Mice were kept under a 12:12 h light:dark schedule, and food and water were available *ad libitum*. Tissue was collected from 5 to 11 litters per age and sex group. Genotypes were confirmed after weaning using tail-tip biopsy and polymerase chain reaction (PCR) amplification (primers: forward 5′-GCTAGCTTGTTATTTATGCTTAAAG-3′; WT reverse 5′-CCACCACACACATGATGCCGAC-3′; *Nrg1* TM HET reverse 5′-GCACAGTCGAGGCTGATCAGCG-3′). Research and animal care procedures were approved by the University of New South Wales Animal Care and Ethics Committee (ACEC) in accordance with the Australian Code of Practice for the Care and Use of Animals for Scientific Purposes (ACEC approval number: 10/98B). The protocol was approved by the University of New South Wales ACEC.

### Tissue Preparation and RNA Extraction

Total RNA was extracted for quantitative PCR (qPCR) analysis from the left hippocampus (4.3–18.3 mg, *N* = 181) and prelimbic cortex (1.3–11.7 mg, *N* = 147) of male and female *Nrg1* TM HET and WT littermates at PND 7, 10, 14, 21, 28, 35, and 161 (Table 1) using TRIzol following the manufacturer’s protocol (Invitrogen, Carlsbad, CA, USA). Total RNA was suspended in DEPC-treated water after purification by precipitation (Sigma-Aldrich, Castle Hill, NSW, Australia). The yield of total RNA was analysed using a spectrophotometer (Nanodrop ND-1000; Thermo Scientific, Wilmington, DE, USA). The quality of total RNA was determined using an Agilent Bioanalyzer 2100 (Agilent Technologies, Palo Alto, CA, USA): 100–200 ng RNA was applied to an RNA 6000 Nano LabChip, without heating prior to loading. The RNA integrity number (RIN) was used as an indicator of RNA quality, ranging from 1 to 10 (lowest to highest quality). Samples had an average RIN of 9.03 ± 0.10 (Table 1). Any sample with a RIN ≤ 6.5 was excluded from qPCR experiments.

**Table 1:** Summary of developmental cohort used for experiments.

Postnatal day	Genotype	Gender	RIN	*n*
**Hippocampus**				

7	12 WT, 10 *Nrg1* TM HET	11 F, 11 M	9.63 ± 0.47	22
10	12 WT, 12 *Nrg1* TM HET	12 F, 12 M	9.70 ± 0.35	24
14	12 WT, 11 *Nrg1* TM HET	12 F, 11 M	9.24 ± 0.33	23
21	12 WT, 12 *Nrg1* TM HET	12 F, 12 M	9.10 ± 0.25	24
28	12 WT, 12 *Nrg1* TM HET	12 F, 12 M	8.88 ± 0.27	24
35	12 WT, 11 *Nrg1* TM HET	12 F, 11 M	8.73 ± 0.36	23
49	10 WT, 11 *Nrg1* TM HET	11 F, 10 M	8.73 ± 0.46	21
161	10 WT, 10 *Nrg1* TM HET	9 F, 11 M	8.77 ± 0.46	20

**Prelimbic cortex**				

7	9 WT, 7 *Nrg1* TM HET	9 F, 7 M	9.61 ± 0.54	16
10	9 WT, 7 *Nrg1* TM HET	8 F, 8 M	9.48 ± 0.57	15
14	9 WT, 8 *Nrg1* TM HET	10 F, 7 M	9.30 ± 0.53	17
21	9 WT, 7 *Nrg1* TM HET	9 F, 7 M	8.61 ± 1.09	16
28	12 WT, 11 *Nrg1* TM HET	11 F, 12 M	8.75 ± 0.58	23
35	12 WT, 10 *Nrg1* TM HET	11 F, 11 M	8.60 ± 0.76	22
49	9 WT, 8 *Nrg1* TM HET	7 F, 10 M	8.55 ± 0.51	17
161	11 WT, 9 *Nrg1* TM HET	9 F, 11 M	8.81 ± 0.31	20

*F, female; M, male; n, number of mice per postnatal age group; RIN, RNA integrity number*.

### Reverse Transcription and qPCR

Complementary deoxyribonucleic acid (cDNA) was synthesised in two reactions of 0.5–2 µg of total RNA using the Superscript III First-Strand Synthesis Kit (Invitrogen) according to the manufacturer’s protocol. Predesigned TaqMan Gene Expression Assays (Applied Biosystems, Foster City, CA, USA) were chosen for genes of interest and for three housekeeper control transcripts (Table 2). qPCR was performed with an ABI Prism 7900HT Fast real-time PCR system with a 384-well format. The PCR reaction was initiated by uracil–DNA glycosylase treatment for 2 min at 50°C and denaturation for 10 min at 95°C, followed by 40 cycles consisting of heating to 95°C for 15 s followed by annealing and extension at 60°C for 1 min. Measurements were performed in duplicate and relative quantities determined from a seven-point standard curve. Control wells containing no cDNA template displayed no amplification. Efficiencies of the qPCR reactions ranged from 61 to 92%, and *r*^2^ values were between 0.96 and 1.00. Outliers were excluded from qPCR analysis if their normalised expression values were greater than 2.5 SDs from the group mean. Expression levels were normalised to the geometric mean of three reference genes (Table 2).

**Table 2:** Applied Biosystems TaqMan gene expression assay numbers.

Gene name	Gene symbol	Taqman assay
**Genes of interest**		

Cannabinoid CB_1_ receptor	Cnr1	Mm00432621_s1
Diacylglycerol lipase alpha	Daglα	Mm00813830_m1
Monoglyceride lipase	Mgll	Mm00449274_m1
α/β-hydrolase domain containing 6	Abhd6	Mm00481199_m1

**Reference genes**		

TATA box binding protein	Tbp	Mm00446973_m1
Ubiquitin C	Ubc	Mm01201237_m1
Eukaryotic 18S rRNA	18 S	Hs99999901_s1

### Statistical Analysis

Quantitative PCR data were analysed using SPSS Statistics 22 (IBM, NY, USA). Differences in normalised mRNA expression were assessed with three-way analysis of variance (ANOVA) with “genotype” (WT/*Nrg1* TM HET), “sex” (male/female), and “age” (PND 7, 10, 14, 21, 28, 35, 49, and 161) as between factors. Polynomial contrasts were used to identify linear, quadratic, and cubic trends in mRNA expression across PND ages. Data are presented as mean ± SEM, and differences were regarded as statistically significant if *p* < 0.05.

## Results

### Housekeeper Controls

The expression of house-keeping genes was stable across postnatal development. The geometric mean of the expression of Ubc and Tbp mRNA and 18S rRNA did not change significantly over postnatal development in the hippocampus or prelimbic cortex (Figure S1 in Supplementary Material).

### Sex Differences

Endocannabinoid mRNA expression did not differ between male and female mice in either brain region for any endocannabinoid marker investigated; thus, data were collapsed across sex (three-way ANOVA main effect of “sex”: all *F* < 1, all *p* > 0.05).

### Cannabinoid Receptor 1

**Figure 1:**
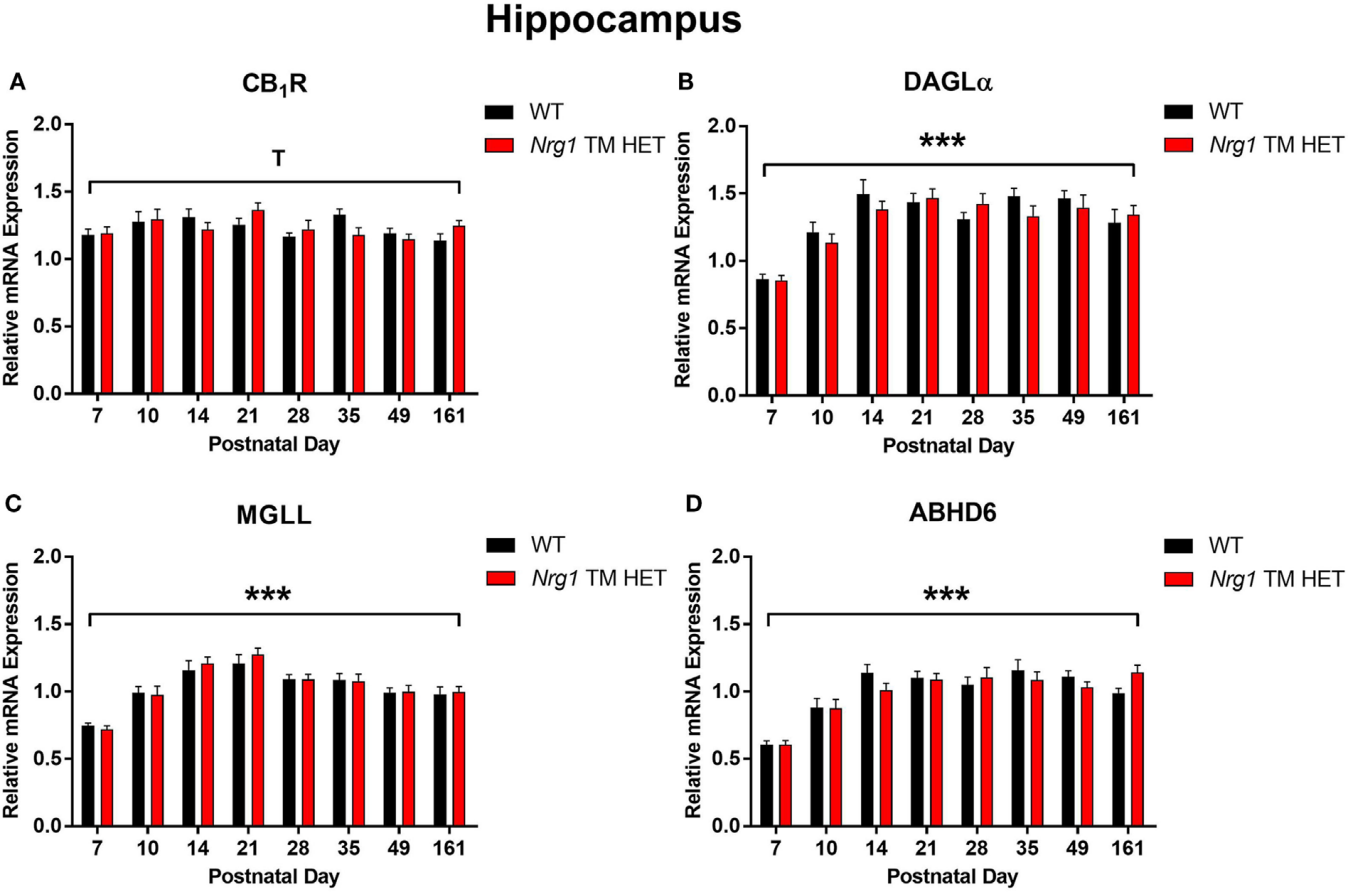
Endocannabinoid mRNA expression in hippocampus of heterozygous transmembrane domain *Nrg1* mutant mice (*Nrg1* TM HET mice) and wild type (WT)-like controls. Expression of mRNA for **(A)** CB_1_ receptor (CB_1_R), **(B)** diacylglycerol lipase alpha (DAGLα), **(C)** monoglycerol lipase (MGLL), and **(D)** α/β-hydrolase domain-containing 6 (ABHD6) was determined by quantitative polymerase chain reaction [*y*-axis, mean (±SEM) expression normalized to the geometric mean of three reference genes] and plotted by postnatal day. Main effects of “age” are indicated on graphs by asterisks (***p < 0.001); trends for effects of age are indicated by “T” (^T^*p* = 0.06).

**Figure 2:**
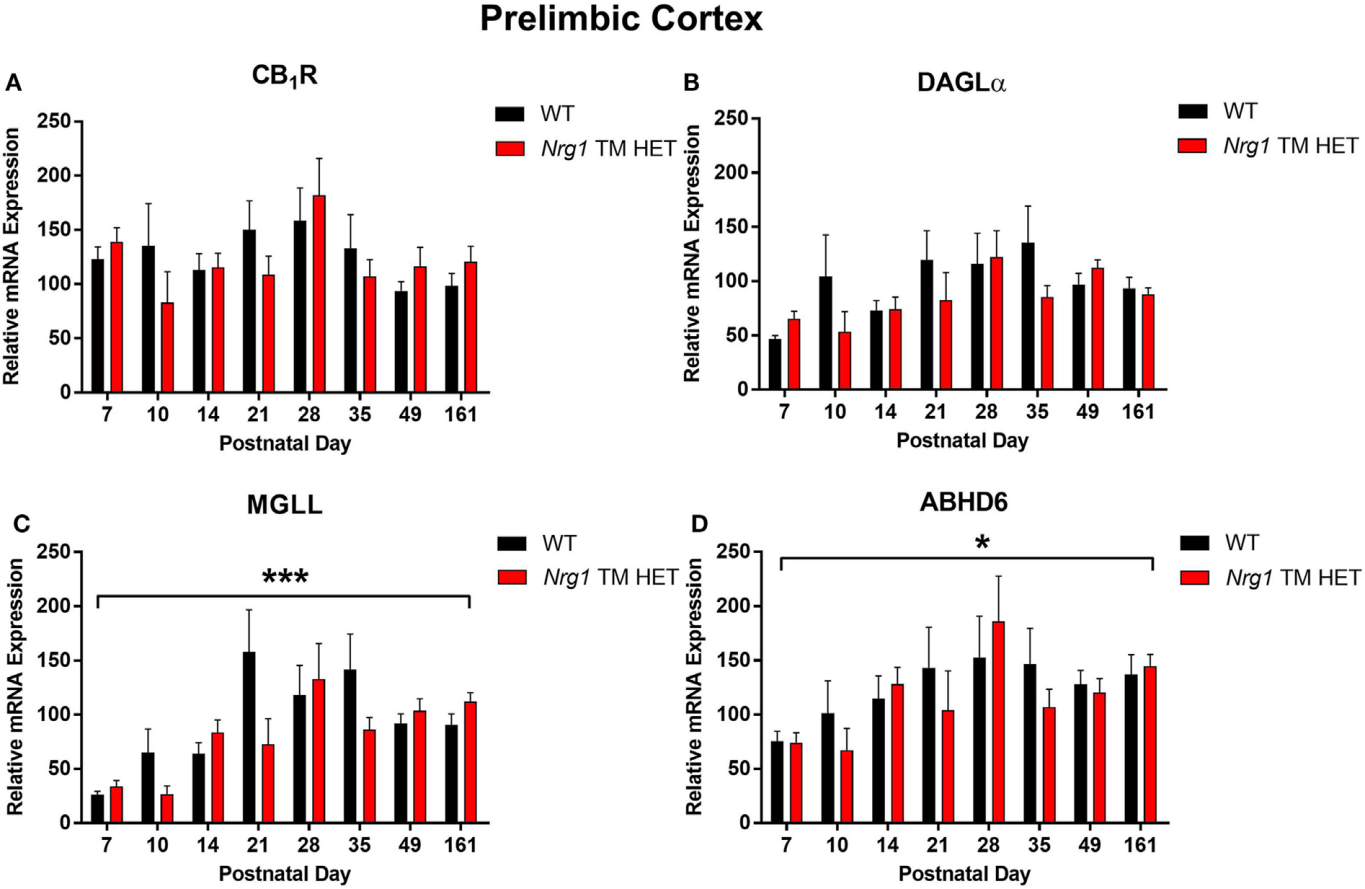
Endocannabinoid mRNA expression in the prelimbic cortex of heterozygous transmembrane domain *Nrg1* mutant mice (*Nrg1* TM HET mice) and wild type (WT)-like controls. Expression of mRNA for **(A)** CB_1_ receptor (CB_1_R), **(B)** diacylglycerol lipase alpha (DAGLα), **(C)** monoglycerol lipase (MGLL), and **(D)** α/β-hydrolase domain-containing 6 (ABHD6) was determined by quantitative polymerase chain reaction [*y*-axis, mean (±SEM) expression normalized to the geometric mean of three reference genes] and plotted by postnatal day. Main effects of “age” are indicated on graphs by asterisks (*p < 0.05, ***p < 0.001).

Cannabinoid receptor 1 mRNA expression exhibited a trend for changes across development in the hippocampus, but not in the prelimbic cortex [main effect of “age”: hippocampus *F*(7,158) = 2.0, *p* = 0.06; Figure 1A; prelimbic cortex *F*(7,134) = 1.6, *p* = 0.10; Figure 2A]. A significant quadratic contrast (*p* = 0.047) for hippocampal CB_1_R mRNA expression data indicates that overall CB_1_R expression increased until PND 21 and decreased after that age. There were no differences in CB_1_R mRNA expression between WT and *Nrg1* TM HET mice in the hippocampus [*F*(1,158) = 0.01, *p* = 0.90] or prelimbic cortex [*F*(1,134) = 0.1, *p* = 0.80].

### Diacylglycerol Lipase Alpha

Diacylglycerol lipase alpha mRNA expression changed significantly during postnatal development in the hippocampus [main effect of “age”: *F*(7,158) = 15.5, *p* < 0.001; Figure 1B], with a significant cubic contrast (*p* = 0.004), indicating that DAGLα mRNA expression increased from PND 7 to 21, then decreased at PND 28, and stabilised for the rest of postnatal development. Hippocampal DAGLα mRNA expression was not different between WT and *Nrg1* TM HET mice across postnatal development [*F*(1,158) = 0.60, *p* = 0.50]. There was no significant change in postnatal DAGLα mRNA expression across development in the prelimbic cortex overall [*F*(7,134) = 1.7, *p* = 0.10]; this was not different between WT and *Nrg1* TM HET mice [*F*(1,134) = 1.2, *p* = 0.30; Figure 2B].

### Monoglyceride Lipase

Monoglyceride lipase mRNA expression changed significantly during postnatal development in both the hippocampus and the prelimbic cortex [hippocampus: *F*(7,158) = 19.1, *p* < 0.001; prelimbic cortex: *F*(7,135) = 4.8, *p* < 0.001]. In the hippocampus, MGLL mRNA expression increased from PND 7 to 14, then dropped, and remained stable until PND 35, before dropping to a lower level of expression at PND 49–161 (significant cubic contrast, *p* < 0.001; Figure 1C). In the prelimbic cortex, MGLL mRNA expression increased in a linear fashion over postnatal development, reaching peak levels at PND 35 (significant linear contrast, *p* < 0.001; Figure 2C). In both the hippocampus and the prelimbic cortex, MGLL mRNA expression across development was similar in WT and *Nrg1* TM HET mice [hippocampus: *F*(1,158) = 0.2, *p* = 0.70; prelimbic cortex: *F*(1,134) = 1.4, *p* = 0.20].

### α/β-Hydrolase Domain-Containing 6

α/β-Hydrolase domain-containing 6 mRNA expression levels showed significant developmental changes in both brain regions investigated [hippocampus: *F*(7,157) = 19.6, *p* < 0.001; prelimbic cortex *F*(7,132) = 2.5, *p* = 0.02]. In the hippocampus, ABHD6 mRNA expression slowly increased until PND 14–21, after which it stabilised (significant quadratic contrast, *p* < 0.001; Figure 1D). In the prelimbic cortex, ABHD6 mRNA expression increased in a linear fashion over postnatal development until PND 28 (significant linear contrast, *p* = 0.006; Figure 2D). ABHD6 mRNA expression was not different between WT and *Nrg1* TM HET mice in either brain region investigated [hippocampus: *F*(1,157) = 0.10, *p* = 0.70; prelimbic cortex: *F*(1,132) = 0.40, *p* = 0.50].

## Discussion

The present experiments describe in detail the postnatal development of the transcripts encoding CB_1_R and 2-AG metabolic enzymes DAGLα, MGLL, and ABHD6 in control (WT) and transmembrane domain *Nrg1* mutant mice. There was a trend for postnatal correlation of hippocampal CB_1_R mRNA with age, and an analysis of non-linear change over time detected increased hippocampal CB_1_R expression until PND 21. We found robust support for the developmental alterations in transcripts involved in the regulation of 2-AG in the hippocampus and prelimbic cortex. Expression of the enzymes catalysing 2-AG synthesis (DAGLα) and hydrolysis (MGLL and ABHD6) increased markedly in the first few weeks of postnatal life, with peak levels being reached in the hippocampus at PND 14, about 2 weeks prior to the prefrontal cortex (PND 28–35). We did not find any evidence that inheriting mutant *Nrg1* leads to altered endocannabinoid-related mRNA levels: *Nrg1* TM HET mice displayed similar developmental trajectories of endocannabinoid markers as WT mice.

We found changes in CB_1_R mRNA expression in the hippocampus across postnatal development: CB_1_R mRNA expression increased from PND 7 to 21 and decreased thereafter. A similar pattern of hippocampal CB_1_ mRNA expression has been reported in rats; CB_1_ mRNA expression peaks in early postnatal development and decreases in adulthood (34). Our findings also correspond with reports using human hippocampal tissue; higher CB_1_ mRNA expression is present in fetal human hippocampal tissue (approximately 20 weeks into development) compared to adult hippocampal tissue (46–62 years old) (35). The increase in CB_1_R mRNA expression in early postnatal development may correspond with the regulation of neurotransmission by the endocannabinoid system in early life. The reduction in CB_1_R mRNA expression following PND 21 may be linked to the maturation of GABAergic hippocampal interneurons (36), which take over inhibitory presynaptic neurotransmission previously regulated by the endocannabinoid system (17).

Prelimbic CB_1_R mRNA expression did not change across postnatal development. This may be because non-human cortical CB_1_R mRNA expression has been found to be more dynamic during embryonic than postnatal development. Rats exhibit highest CB_1_R mRNA ratios in the cerebral cortex during gestational and early postnatal development (PND 1 and PND 5) (34, 37), and in macaques, dynamic changes in DLPFC CB_1_R mRNA are detected predominantly during embryonic development (38). Thus, measuring mRNA expression from PND 7 onwards may have been too late to detect substantial changes in cortical CB_1_ mRNA expression.

This study was not able to detect changes in CB_1_R mRNA expression in *Nrg1* TM HET mice compared to WT littermates. Previous autoradiographic receptor binding studies in the same mouse line demonstrated decreased CB_1_R protein levels in the substantia nigra in adolescence (PND 53) and increased levels in adulthood (PND 98–140) (10, 28). However, CB_1_R binding in the medial prefrontal cortex and hippocampus in adult *Nrg1* TM HET was not different to that of WT mice (10, 28), which is in line with our findings. This suggests region-specific changes in CB_1_R expression in *Nrg1* TM HET mice. It is also possible that there are differences between the expression profiles of CB_1_R receptor protein and mRNA levels, similar to what has been observed in human schizophrenia DLPFC tissue (39, 40).

We observed dynamic changes in mRNA expression of DAGLα, MGLL, and ABHD6 across postnatal development in both the hippocampus and the prelimbic cortex. Our study is the first to describe mouse ABHD6 mRNA expression across postnatal development; this has not been reported in rodents or non-human primates before. The developmental expression trajectory of prelimbic DAGLα and MGLL reported here agrees with the study by Subbanna et al. describing a similar increase and stabilisation of DAGLα and MGLL protein expression in the neocortical tissue of C57BL/6J mice across postnatal development (41). DAGLα protein expression increased from PND 2 and peaked at PND 15 in the neocortex, while MAGL protein expression increased from PND 2 and peaked at PND 45 (41). These peaks in 2-AG metabolic enzyme mRNA levels may represent periods of high endogenous cannabinoid system activity during postnatal brain development (42).

Importantly, our mouse DAGLα and MGLL mRNA expression profiles show similarities with human trajectories. We observed pronounced early postnatal increases in DAGLα and ABHD6 mRNA expression in the prelimbic cortex between PND 7 and 35, which decreased slightly and then stabilised after this age. Mouse PND 7–35 corresponds with a human age range of infant to 11-year-old children (43), and this pattern of DAGLα and ABHD6 mRNA expression is largely consistent with expression patterns in human DLPFC tissue (17). Furthermore, a reduction in MGLL mRNA expression after puberty occurs in humans in the DLPFC (17), which we also observed in mice in the prelimbic cortex (*cf*. PND 35 vs. PND 161). This may suggest similar regulation of these enzymes across development in the two species and is important when evaluating the validity of mouse model research in this context. It is possible that the increase in enzyme mRNA expression in early postnatal life reflects the involvement of the endocannabinoid system in neuronal growth and migration (44, 45), while its stabilisation after PND 14 (hippocampus) or PND 35 (prelimbic cortex) may represent a switch to mediating retrograde signalling, whereby chemical messengers released from the postsynaptic cell bind to the presynaptic cell to mediate cell signalling. The involvement of the endocannabinoid system in retrograde signalling then appears to remain unchanged for the rest of postnatal life (46), which may be reflected in the reduction and stabilisation of these transcripts after PND 14 or 35.

Our findings suggest different rates of endocannabinoid system maturation of the prelimbic cortex and the hippocampus. The earlier peak in the hippocampus, compared to the prelimbic cortex, may indicate a longer period of maturation of the latter region (47). Indeed, the role of the endocannabinoid system in the stabilisation of cortical excitatory synapses, the balancing excitatory and inhibitory neurotransmission, and strengthening or elimination of excitatory cortical synapses during adolescence (48) support the extended maturation of this system in the prelimbic cortex, compared to the hippocampus. It is possible that fluctuations in endocannabinoid gene expression in early postnatal development (i.e., up to PND 14 or 35), which corresponds with early adolescence in humans, indicate a period during which this system is more susceptible to cannabinoid-induced developmental disruption, which may also increase the risk of cannabinoid-induced psychosis.

There were no differences in DAGLα, MGLL, or ABHD6 mRNA expression in *Nrg1* TM HET mice across development, compared to WT mice. This is the first study investigating these markers across development in a genetic mouse model for schizophrenia. Evidence for altered DAGLα, MGLL, or ABHD6 mRNA expression in schizophrenia is mixed: elevated ABHD6 mRNA expression in PFC (Brodmann area 9) has been observed in patients with an illness duration of less than 15 years (49), whereas no differences in MGLL and DAGLα mRNA expression were found in a different cortical region, the DLPFC (Brodmann area 46) of patients with schizophrenia (50). In both of these studies, it is unknown whether these patients carried *NRG1* risk alleles. It is possible that changes in 2-AG enzyme function may play a role in the development of schizophrenia-related phenotypes; however, this does not appear to depend on mutation in the *NRG1* TM gene.

Heterozygous transmembrane domain *Nrg1* mutant mice exhibit altered sensitivity to cannabinoid compounds (23–28), which, based on this study, appears unrelated to changes in the hippocampal or prelimbic cortical CB_1_ or 2-AG enzyme mRNA expression levels. It is possible that alterations to the endocannabinoid system are present only in *Nrg1* TM HET mice following a cannabinoid challenge to this system [e.g., Ref. (27, 28)]. Alternatively, alternate signalling pathways and/or receptor systems may be responsible for cannabinoid-induced behavioural and neural changes in *Nrg1* TM HET mice. *Nrg1* TM HET mutation may cause alterations to other neurotransmitter systems that interact with the cannabinoid system, thereby mediating increased cannabinoid sensitivity. For example, serotonin 2A receptors (5-HT_2A_) and CB_1_ receptors form heteromers, and cross-antagonise each other at a receptor level to mediate behavioural effects of THC (51, 52). Importantly, our own work shows that *Nrg1* TM HET mice exhibit some region-dependent reductions in 5-HT_2A_ receptor binding (27, 28), but increased 5-HT_2A_ binding across the striatum and frontal cortex (53). Thus, it is possible that altered 5-HT_2A_ receptor binding may affect how cannabinoid compounds bind to CB_1_R and thereby change the behavioural response of *Nrg1* TM HET mice to cannabinoid treatment.

Alternatively, CB_1_ receptors may interact with *N*-methyl-d-aspartate (NMDA) receptors to modulate responses to cannabinoids in *Nrg1* TM HET mice. CB_1_ and NMDA receptors interact to regulate cortical excitatory signalling (54). Also, adolescent THC treatment elevates NMDA receptor expression in the hippocampus, as well as auditory and insular cortices in *Nrg1* TM HET, but not WT mice, supporting potential interactions between glutamatergic and cannabinoid systems in *Nrg1* mutant mice. Considering the dynamic nature of NMDA receptor development [e.g., changes in NMDA receptor expression on fast-spiking interneurons during cortical development (55) and elevated *c-fos* expression following NMDA receptor antagonist MK801 treatment, during early, but not late adolescence (56)], it is possible that baseline NMDA receptor expression may be altered in *Nrg1* TM HET mice; this may affect CB_1_ receptor signalling to modulate behavioural and neural responses to cannabinoids in these mice.

In conclusion, this study demonstrates dynamic postnatal regulation of transcripts encoding key molecules of the endocannabinoid system. Our detailed mRNA expression profile of 2-AG enzymes is in line with the idea that the endocannabinoid system changes as the brain develops. Critically, these data highlight potential key postnatal time points where endocannabinoid system maturation may be susceptible to environmental insults, which could influence the development of mental illness. The mRNA transcripts examined were unaltered in *Nrg1* TM HET mice, suggesting that these mice may not represent a model of altered endocannabinoid development.

## Ethics Statement

Research and animal care procedures were approved by the University of New South Wales Animal Care and Ethics Committee (ACEC) in accordance with the Australian Code of Practice for the Care and Use of Animals for Scientific Purposes (ACEC approval number: 10/98B). The protocol was approved by the University of New South Wales Animal Care and Ethics Committee.

## Author Contributions

LL, RC, CW, and TK designed the research; LL performed the research; RC completed the data analysis and wrote the manuscript; and LL, RC, CW, and TK revised the manuscript and approved it prior to submission.

## Conflict of Interest Statement

The research was conducted in the absence of any commercial or financial relationships that could be construed as a potential conflict of interest.

## Abbreviations

2-AG, 2-arachidonoylglycerol; ABHD6, alpha/beta-hydrolase domain-containing 6; AEA, *N*-arachidonoylethanolamine; ANOVA, analysis of variance; CB_1_R, cannabinoid receptor 1; cDNA, complementary deoxyribonucleic acid; DAGLα, diacylglycerol lipase alpha; DLPFC, dorsolateral prefrontal cortex; MGLL, monoglyceride lipase; mRNA, messenger ribonucleic acid; Nrg1 TM HET, Neuregulin 1 transmembrane domain heterozygous; PND, postnatal day; qPCR, quantitative polymerase chain reaction; RIN, RNA integrity number; RNA, ribonucleic acid; SEM, standard error of the mean; WT, wild type.
