# Supplementary material for: The Endocannabinoid System across Postnatal Development in Transmembrane Domain Neuregulin 1 Mutant Mice
Source: Front Psychiatry. 2018 Feb 7;9:11. doi: 10.3389/fpsyt.2018.00011 (PMC5808294; doi:10.3389/fpsyt.2018.00011)
Supplement: Supplementary file 1 [file Image_1.PDF]

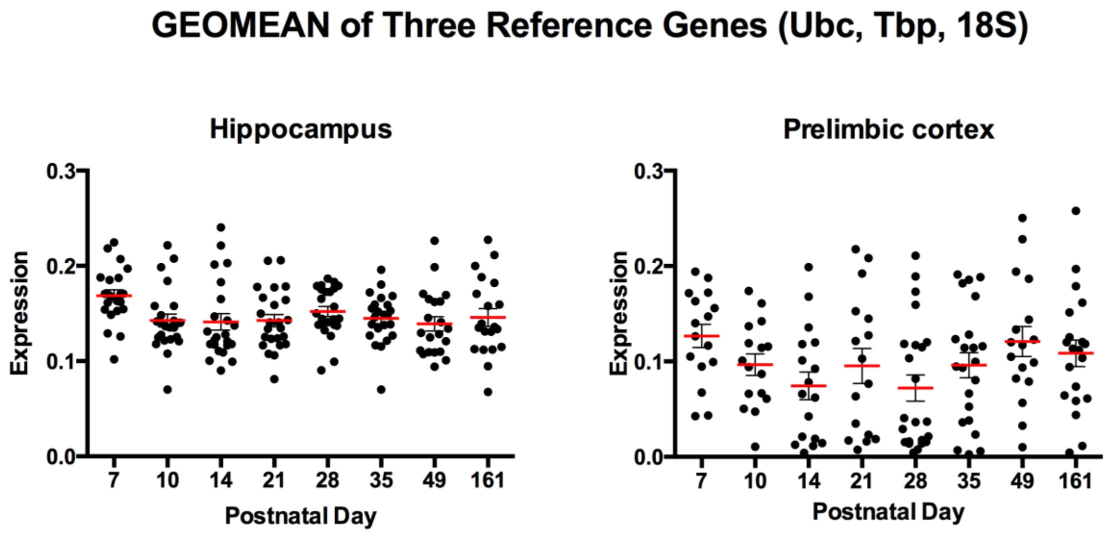

Supplementary figure 1. Geometric mean house of expression of housekeeping genes. Geometric mean of expression of Tbp and Ubc mRNA and 18S rRNA in hippocampus and prelimbic cortex of Nrg1 TM HET mice and WT-like controls determined by qPCR (y-axis, mean ± SEM expression) plotted by postnatal day. n = 16-24.
